# Serial decision-making and noise in the assembly of neural circuits

**DOI:** 10.1186/1471-2202-12-S1-O11

**Published:** 2011-07-18

**Authors:** Aldo A Faisal, Victor A Luria

**Affiliations:** 1 Department of Bioengineering, Imperial College London, London, SW7 2AZ, UK; 2 Department of Computing, Imperial College London, London, SW7 2AZ, UK; 3 Department of Genetics, Columbia University, New York (NY), USA; 4 Motor Neuron Center, Columbia University, New York (NY), USA

## 

Accurate decision-making is essential for the correct assembly of sensory-motor circuits, which are composed of neurons whose axonal growth cones execute discrete, binary decisions at sequential trajectory selection points to connect motor neurons to target muscles. Limb motor axon trajectory selection is controlled by guidance cues: ephrin ligands and Eph receptors [1], whose expression levels are variable. Some cues direct axons to opposite trajectories. Genetic inactivation of Eph and ephrin cues results in inaccurate trajectory decisions and incorrect neural circuit topology [2]. To understand this decision making process we developed a model of an axon growth cone as it grows from the spinal chord,passing binary decision regions to innervate its target muscle. Our model linearly sums noisy guidance cues at receptors on the growth cone surface, which in turn drive cytoskeletal dynamics of their nearest-neighbour microtubule (resulting in the protraction/ of individual filopodia of the growth cone). Our simple model incorporates three basic constraints: 1. guidance cues are noisy signal due to gene expression variability and stochastic ligand-receptor interactions, 2. guidance cue combination is linear and additive and, 3. raw material constraints during growth, limit the total size of the growth cone. Combining these basic constraints suggests a decision-making model that explains in a unified way 4 experimental findings:

First, at binary decision points genetically homogenous populations of axons can partition on the two possible trajectories tissues with an unequal ratio. Unequal ratios are observed in very different developmental decisions e.g. *Bacillus subtilis* cell fate (80:20), *Drosophila* photoreceptor type(70:30), the optic chiasma of vertebrate retinae(97:3) and in frog, chicken and mouse motor systems(93:7-96:4) [2,3]. It was unclear how such unequal ratios are reliably produced, as deterministic mechanisms for decision-making could account for 100:0, and 50:50 ratios but not intermediate ones. Second, shifts in partition ratios were observed in genetic mutation studies where cues were removed or added, in our ephrin and Eph mouse mutants, these decision were inordinately variable (from 95:5 in wild-type to 80:20, 60:40, 0:100) [2]. Third, axons growing in genetically symmetrised limbs (where dorsal or ventral limb halves are duplicated) grow with equal probability towards either target or fail to enter the limb [4]. Fourth, experiments showed that growth cones faced with decisions slow their movement considerably (500-1,000%) and grow in diameter (300-1000%). Thus, the decision-making machinery grows commensurately in size and sensing capacity (receptor-covered axon surface area). Our model reproduces this as the repulsive cues for each target competitively engage cytoskeletal material towards targets and reducing availability of material devoted to forward movement. Once decisions are made, our model growth cone shrinks as cytoskeletal material is used for rapid axon growth in accordance with experiments.

In conclusion, we developed a mechanistic model of serial decision-making in axonal growth cones that explains all four experimental findings. Our findings highlight the axons and generally neurons during circuit assembly in development, face computational problems that are comparable to decision making under uncertainty in whole animals, such as sensory processing, decision-making and control of movement [5].
